# 18S rDNA dataset profiling microeukaryotic populations within Chicago area nearshore waters

**DOI:** 10.1016/j.dib.2015.12.042

**Published:** 2016-01-07

**Authors:** Daniel Searle, Emily Sible, Alexandria Cooper, Catherine Putonti

**Affiliations:** a The Woodhall School, 58 Harrison Lane, Behlehem, CT 06751, USA; b Department of Biology, Loyola University Chicago, 1032 W Sheridan Rd, Chicago, IL 60660, USA; c Bioinformatics Program, Loyola University Chicago, 1032 W Sheridan Rd, Chicago, IL 60660, USA; d Department of Computer Science, Loyola University Chicago, 820 N Michigan Ave, Chicago, IL 60611, USA

**Keywords:** Microeukaryotic community, Metagenomics, Freshwater, Lake Michigan

## Abstract

Despite their critical role in the aquatic food web and nutrient cycling, microeukaryotes within freshwater environments are under-studied. Herein we present the first high-throughput molecular survey of microeukaryotes within Lake Michigan. Every two weeks from May 13 to August 5, 2014, we collected surface water samples from the nearshore waters of four Chicago area beaches: Gillson Park, Montrose Beach, 57th Street Beach, and Calumet Beach. Four biological replicates were collected for each sampling date and location, resulting in 112 samples. Eighty-nine of these samples were surveyed through targeted sequencing of the V7 and V8 regions of the 18S rDNA gene. Both technical and biological replicates were sequenced and are included in this dataset. Raw sequence data is available via NCBI’s SRA database (BioProject PRJNA294919).

**Specifications Table**

**Table:** 

Subject area	*Biology*
More specific subject area	*Microeukaryotic metagenomics*
Type of data	*Text files: sequences*
How data was acquired	*Illumina MiSeq Desktop Sequencer*
Data format	*Raw*
Experimental factors	*DNA extracted from microeukarytoic cells captured using 0.45μm filters.*
Experimental features	*Amplification of the V7 and V8 regions of the 18 S rDNA gene. Sequencing using the MiSeq Reagent Kit v2 (500-cycles) kit for the Illumina MiSeq platform.*
Data source location	Chicago, IL, USA: Montrose Beach (41°58′0.71″N, 87°38′13.35″W), 57^th^ Street Beach (41°47′25.54″N, 87°34′41.25″W), and Calumet Beach (41°43′8.18″N, 87°31′32.51″W); Wilmette, IL, USA: Gillson Park (42°4′45.10″N, 87°40′59.10″W).
Data accessibility	*Raw data is available through NCBI*׳*s BioProject database,* Accession*: PRJNA294919 (http://www.ncbi.nlm.nih.gov/bioproject/PRJNA294919).*

**Value of the data**

• This is the first broad, high-throughput inquiry of microeukaryotic species from freshwater nearshore waters within the Great Lakes region.
• The data provide a survey of microeukaryotic diversity within urban freshwaters during the summer months.
• The inclusion of biological replicates within the dataset documents putative microeukaryotic diversity within sites.
• While microeukaryotic surveys can target a variety of genetic markers, the data presented here can serve as a benchmark for the breadth and resolution of taxonomical classification possible by the 18S V7 and V8 regions in complex environmental communities.

## 1 Experimental design, materials and methods

### 1.1 Sample collection

The nearshore waters of four Chicago area beaches were sampled: Gillson Park (42°4′45.10″N, 87°40′59.10″W), Montrose Beach (41°58′0.71″N, 87°38′13.35″W), 57th Street Beach (41°47′25.54″N, 87°34′41.25″W), and Calumet Beach (41°43′8.18″N, 87°31′32.51″W). Surface water was collected in sterile polypropylene bottles (4 L capacity) at a distance from the shore such that the water level was approximately 0.5 m deep. No specific permits or permissions were required for the water samples collected from the Chicago nearshore waters; a permit was obtained for Gillson Park in accordance with the Wilmette Park District. For each site, four replicates (within a 5 m area) were collected every two weeks between May 13 and August 5, 2014. In total, 112 samples were taken.

### 1.2 Microeukaryotic Isolation

The water was filtered through sterile 0.45 μm bottle-top cellulose acetate membrane filters (Corning Inc., Corning, NY) to capture plant matter, sand, debris, and eukaryotic cells. While 16 samples containing high concentrations of mineral and organic solids were processed using two filters (2 L each), the other samples were processed through a single filter. The filter paper was removed from the bottle-top filter using a sterile scalpel and forceps. The filter membrane was then placed into a sterile petri dish. Each filter was then cut into small (1 cm^2^) pieces and promptly processed for DNA extraction.

### 1.3 DNA extraction

DNA was extracted using the MO BIO Laboratories PowerSoil® DNA Isolation Kit (Carlsbad, CA). Six to eight filter membrane pieces were added to the PowerBead Tubes in the kit. The protocol recommended by the manufacturer was followed with the exception of an extended disruption step (15–30 min). DNA was confirmed via agarose gel (1.2%) as well as by the Qubit® Fluorometer (Life Technologies, Carlsbad, CA). DNA was stored at −20 °C until sequencing.

### 1.4 18S rDNA amplification

Eukaryotic 18S and universal 16S/18S rDNA primers [1] were tested against samples collected from the nearshore waters, as well as against *Saccharomyces cerevisiae* DNA (serving as a control). The EUK1181 (5′-TTA ATT TGA CTC AAC RCG GG-3′) and EUK1624 (5′-CGG GCG GTG TGT ACA AAG G-3′) primers were selected; these primers produce an amplicon ~444 bp. As shown in Wang et al. [1], both primers are expected to “cover” much of the cataloged eukaryotic diversity. The aforementioned primer sequences with the appended Illumina adapter overhang nucleotide sequences were obtained from MWG Operon (Huntsville, AL). Amplification was performed as follows: 0.5 μL of each primer (100 mM concentration), 2 μL of extracted DNA, 25 μL of Ready PCR Mix (Amresco, Solon, OH), and 22 μL of nuclease free water. Each reaction was amplified as follows: initial denaturing at 94 °C for 5 min, thirty-five cycles of 94 °C for 30 s, 50.3 °C for 30 s, and 72 °C for 1 min, followed by a final extension at 72 °C for 5 min. Amplification was verified via gel electrophoresis in a 1.2% agarose gel. Positive (*S. cerevisiae*) and negative controls (nuclease free water) were also amplified following the same procedure. The resulting PCR products were then purified using the E.Z.N.A. cycle pure kit (Omega Bio-Tek Inc., Norcross, GA) according to the manufacturer׳s instructions.

### 1.5 Index PCR

To facilitate multiplexing, each PCR product was subsequently amplified again using primers including the Illumina adapter sequences and indexing sequences for subsequent de-multiplexing. Samples were multiplexed using the NEBNext® Multiplex Oligos for Illumina® (Dual Index Primers Set 1) (New England Biolabs, Ipswich, MA). Subsequent DNA preparation – PCR clean-up, library pooling, and sample loading – followed the standard protocols established by Illumina for the MiSeq Benchtop Sequencer [2]. Sequencing was performed using the Illumina MiSeq Benchtop Sequencer (Loyola University Chicago’s Center for Biomedical Informatics, Maywood, IL). Paired end reads, each 250 nucleotides in length, were produced using the Illumina MiSeq Reagent Kit v2 (500-cycles).

### 1.6 Sequence demultiplexing

Demultiplexing of the sequence data was automated by the Illumina sequencer’s CASAVA package.
